# Friend recollections, and a collection of collaborations with Nadia

**DOI:** 10.3389/fnbeh.2022.954906

**Published:** 2022-07-29

**Authors:** Marie-H. Monfils, Hongjoo J. Lee, Roberto U. Cofresí, Rueben A. Gonzales

**Affiliations:** ^1^Department of Psychology, The University of Texas at Austin, Austin, TX, United States; ^2^Department of Psychological Sciences, University of Missouri, Columbia, MO, United States; ^3^College of Pharmacy, The University of Texas at Austin, Austin, TX, United States

**Keywords:** Nadia Chaudhri, alcohol, Pavlovian conditioning, retrieval + extinction, extinction

## Abstract

In this selective review article, we showcase our collaborations with our colleague, Dr. Nadia Chaudhri. Dr. Chaudhri was an esteemed colleague and researcher who contributed greatly to our understanding of Pavlovian alcohol conditioning. From 2014 to 2019, we collaborated with Nadia. Here, we reflect on our friendship, the work we did together, and the continued impact on the field.

## Introduction

This is a science story. It’s also a story of friends doing science together, and celebration of a great scientist, inspiring human, generous collaborator, and dear friend who left this world too soon—Dr. Nadia Chaudhri. 2009 was a very good year. As Barack Obama was swearing in as President of the United-States, Marie was starting a faculty position at The University of Texas at Austin. 2009 is also when she met Nadia, just as she was also about to start her own faculty position at Concordia University in Montreal. Nadia and Marie met at the Gordon Research Conference on Amygdala Function in Emotion, Cognition and Disease meeting organized that year by Drs. Greg Quirk and Denis Paré. This was a first Gordon meeting for Nadia and Marie. They didn’t know then that they would go on to be collaborators, but they both knew they would be friends. There was much to like about the Gordon meeting: the “getting-lost” hikes, the very tall beds of the on-campus accommodations, which required stealthy acrobatics to get in and out of. The science was strong, and the community of contributors was impressive and humbling. Nadia and Marie talked about their excitement (and anxiety) at starting their respective labs. They promised to invite one another to give talks in their new departments.

## Nadia visits the University of Texas at Austin

Not long after she started at UT Austin, Marie invited Nadia to visit and give a talk. In April 2010, Nadia gave a presentation entitled: “Environmental triggers for relapse to alcoholism: defining the neural circuitry using preclinical models.” Marie still vividly remembers that as she introduced Nadia, she mispronounced her last name: “I am pleased to introduce my colleague and friend, Dr. Nadia Shaudri!” “TChaudhri,” Nadia whispered, “with a hard Ch.” Marie never mispronounced Nadia’s last name from that day onward.

Nadia, as usual, gave a great talk. During her visit, she met a number of colleagues at UT Austin, including Joanne and Rueben. At the time, Rueben planted the seed of collaboration… He came to the podium after Nadia’s talk and said “We should collaborate!”

After Nadia’s visit to UT Austin, Joanne and Marie had embarked on collaborations in which they examined whether an effect Marie had previously discovered while working as a postdoc in Dr. Joseph LeDoux’s lab, retrieval + extinction of fear, could successfully reduce the return of food seeking behavior in an appetitive paradigm, and whether counter-conditioning after an isolated retrieval could more persistently update fear memories than standard counter-conditioning (Monfils et al., 2009; Olshavsky et al., 2013). Marie had previously found that by simply presenting an isolated cue-memory retrieval trial, prior to a standard cue-extinction session, that she could prevent the return of fear in rats (Monfils et al., 2009). Together with Joanne’s lab, they discovered that the retrieval-extinction approach could also prevent the return of food seeking behavior (Olshavsky et al., 2013).

Some ideas need incubation time… For quite a while after Nadia’s visit, Rueben had been thinking about initiating a collaboration to test whether the retrieval + extinction manipulation could attenuate cue-triggered alcohol seeking. The thought was also brewing in Marie’s mind. As you can likely relate, the mind of scientists wanders from idea to idea, but what was needed to set this particular one propelling forward was the catalyst.

## Enters Roberto Cofresí: A student with infectious energy and bridging ideas

Finally, after years of envisioning doing the study, Rueben approached Marie and proposed that they carry it out. In 2014: our collaboration began! You see, a new graduate student in his lab, Roberto Cofresí (the catalyst!), had expressed interest in testing the retrieval + extinction manipulation on conditioned responses to alcohol-predictive cues. While both Rueben and Marie were excited at the prospect of finally embarking on a collaboration, it was immediately obvious to them that they did not quite speak the same language. They needed layers of translation for the project to effectively coalesce. Marie exclaimed: “we need Joanne! AND, we need Nadia, from Montreal!” Marie reached out to Joanne, and scheduled a call with Nadia. Both were immediately interested: a new collaboration was born!

**FIGURE 1:**
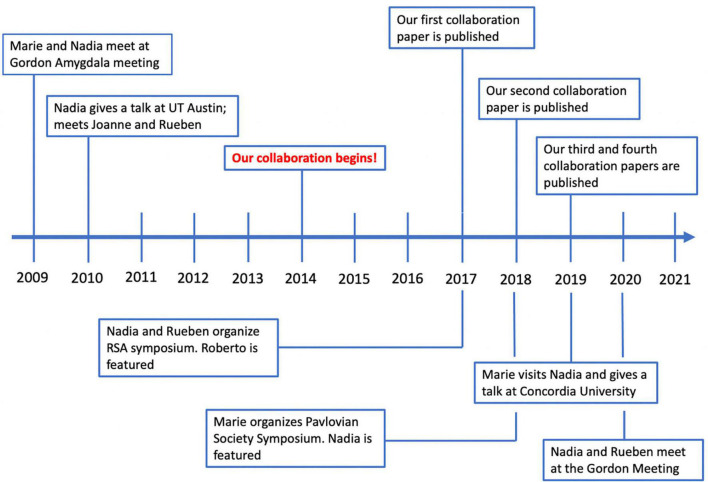
Timeline of events throughout our friendship and collaboration with Nadia.

And thus began our adventure. We regularly chatted on the phone, held meetings online to design experiments (before Zoom was a thing), and discussed preliminary findings along the way. Really, truly, we almost immediately realized that Roberto was not only the catalyst, but also the glue that had long been needed for us to launch this project, and keep it rolling. All-told, over the years that followed, our team completed 4 studies (see Figure 1 for a timeline). Marie still remembers the engaging and animated discussions we all had. And the delighted look on Rueben’s face when Nadia announced to us over a video call that she was pregnant. We were a team that had tremendous fun working together.

In her career, Nadia published extensively and made a significant impact on the field of alcohol research. Here, we showcase the work we carried out together and discuss its continued impact.

## Post-retrieval extinction attenuates alcohol cue reactivity in rats

When individuals drink, cue-associations form, and link pleasurable aspects of drinking with cues in their surroundings: friends, sights, scents, a local bar, music. Repeated episodes of drinking can strengthen the association between alcohol-related cues and turn them into conditioned stimuli for alcohol availability, ingestion, and intoxication (Niaura et al., 1988). As a result, the cues alone can come to capture attention, impel approach, arouse desire for alcohol (“craving”), and induce commensurate physiological changes. Memories evoked by alcohol-associated cues are thus emotionally charged and enduring sources of risk that can precipitate relapse to problematic alcohol use after treatment for alcohol use disorders (Monti et al., 1993; Rohsenow et al., 1994; Papachristou et al., 2014). Cue exposure therapy is a behavioral treatment based on the principles of extinction learning in which the repeated presentation of a conditioned stimulus in the absence of a reinforcer can decrease behavioral, craving, and psychophysiological reactivity to alcohol cues (Staiger and White, 1991; Vollstädt-Klein et al., 2011; Kiefer et al., 2015; MacKillop et al., 2015), decrease self-reported alcohol use quantity and frequency among problem drinkers (Drummond and Glautier, 1994; Rohsenow et al., 2001; Kiefer et al., 2015; MacKillop et al., 2015), and decrease relapse probability and/or severity among individuals treated for alcohol use disorders (Drummond and Glautier, 1994; Sitharthan et al., 1997; Monti et al., 2001; Rohsenow et al., 2001; for counterpoint, see Mellentin et al., 2017). Still, like most behavioral treatments that rely on extinction principles, cue exposure therapy does not preclude the return of behavioral, craving, and psychophysiological reactivity to alcohol cues. The reason for this is that, mechanistically, extinction leads to inhibitory learning that competes for expression with the constellation of behavioral and physiological responses elicited by cues that have been learned to predict alcohol ingestion and intoxication across a lifetime. In other words, cue exposure therapy does not in and of itself diminish the strength of stamped-in cue-alcohol associations.

The retrieval + extinction manipulation, first developed by Marie in Dr. Joseph LeDoux’s lab to decrease the return of fear after conditioning, more persistently reduces fear, and engages brain mechanisms that are different than those engaged during standard extinction (Lee et al., 2006; Monfils et al., 2009; Clem and Huganir, 2010; Tedesco et al., 2014). Joanne and Marie showed that the manipulation could be effective for appetitive memories (Olshavsky et al., 2013). In our first collaboration with Nadia, we wanted to see if retrieval + extinction could do the same for conditioned alcohol cue memories.

Roberto trained male rats to drink in their homecage over the course of 5 weeks. To do so, he introduced 15% unsweetened alcohol to the rats every Monday, Wednesday, and Friday. Next, he conditioned the rats by pairing illumination of the houselight in the conditioning chamber with alcohol availability through a sipper. In the next phase, Roberto employed either standard extinction or retrieval + extinction to reduce the rats’ sipper approach. Finally, he tested the rats in a long-term memory test to see if the sipper approach remained reduced 2 days after the extinction session. Suzanne Lewis, a bright and extremely motivated post-baccalaureate student in Joanne’s lab, worked alongside Roberto and carried out behavior scoring analyses offline, blind to group assignments.

**FIGURE 2:**
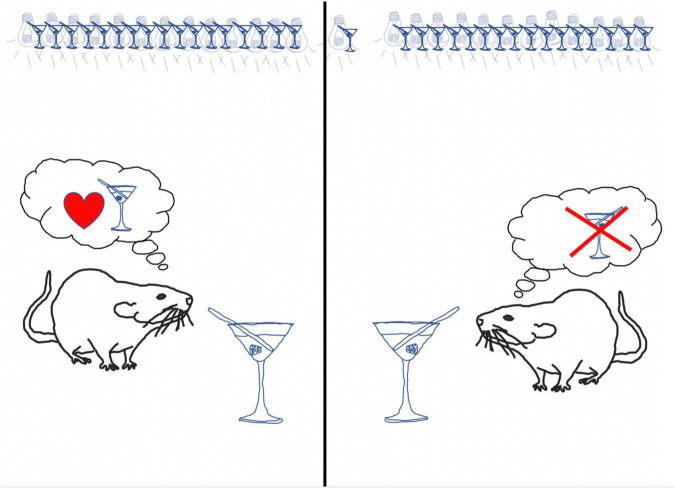
Summary of study on retrieval + extinction for conditioned alcohol cue reactivity. Rats were conditioned to pairings of light and alcohol. Following conditioning, rats received either a standard extinction session, in which the light cue was repeatedly presented in the absence of alcohol, or retrieval + extinction, in which an isolated light cue was presented prior to an extinction session. Following extinction, tests for relapse-like return of conditioned responses to cues previously paired with alcohol were conducted. Conditioned responding to cues previously paired with alcohol readily returned after the passage of time and in response to alcohol odor in the rats that had received standard extinction, but not those that received retrieval + extinction.

We found that the conditioned responding to cues previously paired with alcohol readily returned after the passage of time and in response to alcohol odor in the rats that had received standard extinction, but not those that received retrieval + extinction. Please see Figure 2 for an illustration of the results. These findings are important, because they suggest the possibility of a new treatment tool for alcohol use disorders that may more persistently attenuate alcohol cue reactivity-based risk for relapse than cue exposure therapy.

## Characterizing conditioned reactivity to sequential alcohol-predictive cues in well-trained rats

In our first study described above (Cofresí et al., 2017), we showed that male rats could acquire an inclination to drink alcohol, with the houselight illumination serving as a signal that alcohol would be available through a sipper. In this follow up study (Cofresí et al., 2018), we examined whether the conditioned association extended beyond simply the houselight to include the presentation of the sipper, and the oral receipt of alcohol (as a cue distinct from the ingestion of alcohol and its post-ingestive effects). We wondered if these additional cues would independently exert control over alcohol seeking and drinking, alongside houselight illumination. We also measured blood alcohol levels to ensure that these were commensurate with the amount of drinking the rats’ engaged in, as a validation of our model.

Roberto trained the rats for the homecage drinking and conditioning phases in the same way as in our first study described above. Next, he analyzed sipper approach behavior in exquisite detail by measuring the rats’ behavior before houselight illumination, and during the first and second half of the illumination separately. He also analyzed the latency for the rats to start licking the sipper, and the lick rate across the sipper access window.

We should remind the reader that, in this study, the explicit stimulus that was paired with alcohol access was houselight illumination. Here we wondered if other stimuli present in the task might also acquire predictive value. We found that sipper presentation elicited licking, even when no alcohol was present in the sipper and in the absence of alcohol odor in the air. There was also a strong increase in lick rate upon initial receipt of alcohol delivered orally, suggesting that receiving oral alcohol may, in and of itself, act as a cue that stimulates consummatory behavior.

Taken together, we found that houselight illumination triggered conditioned approach; sipper presentation evoked licking behavior; and intra-oral alcohol receipt promoted drinking. There were individual differences between the rats, with some seeking alcohol more so than others. In additional analyses, we found rats with the greatest conditioned reactivity to the most distal alcohol cue (that is, the cue that was the furthest removed from the alcohol consumption—the houselight) were also the fastest to initiate drinking, and drank the most (Cofresí et al., 2018).

**FIGURE 3:**
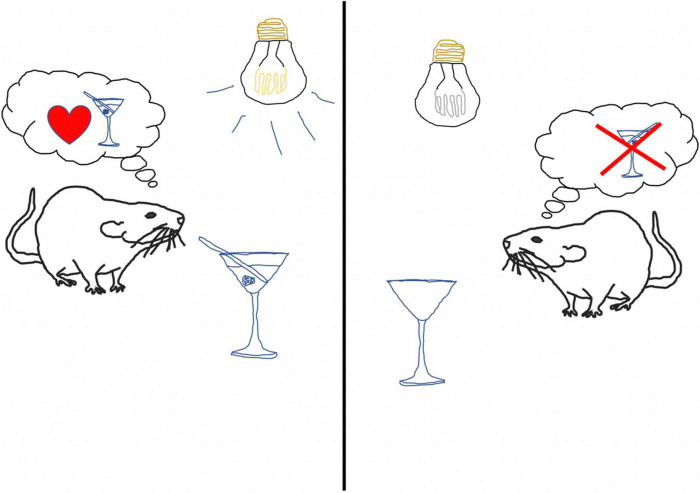
Summary of study characterizing conditioned alcohol cue reactivity. Rats were conditioned to light cues paired with alcohol. We analyzed sipper approach by scoring the rats’ sipper approach behavior before houselight illumination and during the illumination. We also analyzed the latency for the rats to start licking the sipper, and the lick rate across the sipper access window. We found that houselight illumination triggered conditioned approach to the sipper access site. We also found that sipper presentation elicited licking, even when no alcohol was present in the sipper and in the absence of alcohol odor in the air. We also found a strong increase in lick rate upon initial receipt of alcohol from the sipper, suggesting that receiving oral alcohol may, in and of itself, act as a cue that stimulates consummatory behavior (drinking). In additional analyses, we found rats with the greatest conditioned approach reactivity to the most distal alcohol cue (i.e., houselight illumination) also were the fastest to initiate drinking, and drank the most.

These findings are important, because they highlight the fact that alcohol-associated cues are pervasive, and release powerful conditioned responses that can drive the alcohol seeking-drinking cycle. Identifying the cues that can most powerfully trigger alcohol seeking behavior may be clinically useful in developing means of reducing their impact, and as such, decrease cue-based risk for relapse, and helping to improve alcohol use disorder treatment outcomes. Please see Figure 3 for an illustration of the results.

## Alcohol-associated antecedent stimuli elicit alcohol seeking in non-dependent rats and may activate the insula

In our second collaboration, described above, we found that environmental and sensory stimuli present during alcohol ingestion and intoxication can come to act as triggers of alcohol seeking. In that study, however, we did not parse out whether this occurred as a result of mere repeated exposure to alcohol and houselight illumination, or whether it was progressively acquired as a result of associative learning about the contingency between houselight illumination and alcohol availability. We did so in this third study (Cofresí et al., 2019). We also harvested the brains at the end of the study, following a long-term memory test, and processed them for cFos immunohistochemistry to start delineating the brain regions that underlie the cue-alcohol associations that form with repeated ethanol consumption.

Roberto trained the rats similarly to what we described for our first two studies, but this time, he included a group that received unpaired alcohol/houselight conditioning trials—that is, the rats were exposed to houselight illuminations and alcohol availability through the sipper, but the two stimuli never occurred together.

Roberto found that the reactivity to houselight illumination depended on its meaningful association with alcohol. That is, sipper-seeking only occurred in the group in which houselight illumination had been explicitly paired with alcohol availability through the sipper. Importantly, both the paired and unpaired groups actually drank about the same overall throughout the session, because the unpaired rats still reacted to alcohol sipper presentation. However, rats in the two groups showed different reactivity to the sipper and the difference was most obvious at the last training session when the houselight-alcohol association or no association had been fully acquired. Rats in the paired group were faster at initiating licking and showed greater licking intensity at the beginning of the training session, with the vigor of drinking behavior decreasing across trials in the session (i.e., “front-loading” or binge-like drinking behavior). In the unpaired group, the drinking behavior remained steady throughout the training session and ends up surpassing the one in the paired group toward the end of the training session. Taken together, our findings suggest that a progressively formed association between houselight illumination and alcohol leads to our observed changes in behavior across training sessions in our model. In further support of this hypothesis, there were differences in houselight illumination cue-related neural activity between the paired and unpaired groups in the anterior insular cortex—an important region for cue-reward associative memory maintenance (Nasser et al., 2018). Importantly, in our study we could not parse out whether the differences we observed in the insula were due to memory formation or memory expression (Cofresí et al., 2019).

## Cue-alcohol associative learning in female rats

We had previously shown that pairing houselight illumination with alcohol led to associative learning in male rats. Here, we tested whether females could also learn in a similar way. Roberto exposed female rats to alcohol in their homecage on Monday, Wednesday, and Friday, for 5 weeks. Then, he trained them using either our paired cue-alcohol procedure, or an explicitly unpaired version as a control. Interestingly, while the total amount of alcohol ingested by the female rats in this study was larger than we had previously observed in males, the blood alcohol levels were the same (Cofresí et al., 2018, 2019). These findings also corroborated another experiment that Nadia had conducted in her lab (LeCocq et al., 2018). Despite receiving the same number of presentations of houselight illumination and alcohol access, albeit separately, rats that were in the unpaired group did not condition alcohol seeking behaviors to the houselight illumination cue. Other groups had previously shown that females could acquire alcohol conditioning (Sherrill et al., 2011; Torres et al., 2014; Nentwig et al., 2017), but the present study was unique in that it showed that females condition to alcohol cues in a way similar to males, and could have implications as we and other groups develop strategies for reducing cue-triggered alcohol seeking in females (Cofresí et al., 2019).

## Dr. Roberto Cofresí!

On May 24, 2018, Roberto successfully defended his dissertation. Roberto’s dissertation committee members were Nadia, Joanne, Rueben, Marie, as well as Dr. Kim Fromme, a prominent clinical psychologist and alcohol researcher at UT Austin whose human subjects laboratory featured a simulated barroom, experiments in which have illuminated the role of alcohol use-related cues and contexts in shaping the actual vs. perceived effects of alcohol ingestion in humans. Kim helped Roberto connect his graduate work in rodents to major theories and work done with human subjects. She also fostered his long-standing interest in the human side of alcohol and addiction research.

After Roberto’s graduation, he transitioned from working in rats to working with humans, starting a postdoc at the University of Missouri. The 4 collaborators stayed in touch—in large part thanks to Roberto’s regular emails updating us all on his latest discoveries and accomplishments. Despite the miles, Nadia remained a steady supporter and mentor to Roberto. We seized any opportunity to connect over the years, at any of the meetings that we attended. Below are some examples.

## RSA 2014–2019

Throughout our collaboration, Rueben, Roberto, and Nadia. Got see each other in person every year at the meeting of the Research Society on Alcoholism (RSA). Nadia and Rueben would meet to discuss issues related to model validation (e.g., blood alcohol level determination) and model extension (e.g., new paradigms to test the behavioral and neurobiological effects of alcohol cue reactivity). Rueben was so fond of Nadia as a person and a scientist that she always had a seat at Gonzales lab dinner, one of Rueben’s RSA meeting traditions. Nadia also took Roberto under her wing at these meetings, introducing him to other students of behavior working in the alcohol field, relating the history of the field (e.g., about Dr. Jane Stewart’s work on contextual conditioning of alcohol preference and tolerance), and helping him think through ideas for future studies.

One especially memorable meeting for the trio was the 2017 RSA meeting in Denver, CO. At that meeting, Nadia and Rueben co-chaired a symposium on alcohol cue reactivity, focused on advancements in our understanding of its neural mechanisms as well as highlighting new prevention strategies (see Table 1). Nadia’s trainee, Milan Valyear, gave a talk about the role of dopamine in cue-triggered alcohol seeking. It was at Nadia and Rueben’s symposium that we first shared the results of our alcohol cue retrieval + extinction experiment with other alcohol researchers. The symposium attracted a lot of attention. This was Roberto’s first talk at an RSA meeting. He felt excited, but extremely nervous. Nadia helped Roberto refine and practice his talk in the weeks before the meeting, and Roberto’s nervousness vanished the moment Nadia introduced him at the symposium. “The smile on her face and the confidence in her voice had a powerful calming effect on me,” he remembers fondly. Roberto’s talk about the fruits of our collaboration stimulated a lot of discussion at that symposium, including strong words of encouragement from Dr. Peter Monti, one of the pioneers of cue exposure therapy as an alcohol use disorder treatment tool, and Dr. Antonio Noronha, the director of the Division of Neuroscience and Behavior at the National Institute for Alcohol Abuse and Alcoholism (NIAAA).

**TABLE 1:** Symposium organized by Nadia Chaudhri and Rueben Gonzales featuring Milan Valyear, Jocelyn Richard, Roberto Cofresí, Robert Swift, and Rainer Spanagel, presented at the Research Society on Alcoholism Meeting in Denver, Colorado in 2017.

Alcohol cue reactivity: new neural mechanisms and prevention strategies
**Research society on Alcoholism, Denver 2017**
**Introduction**
Nadia Chaudhri; Concordia University, Center for Studies in Behavioral Neurobiology, Montreal, QC, Canada
**Cue-elicited alcohol-seeking behavior: zooming in on striatal dopamine**
Milan Valyear; Concordia University, Center for Studies in Behavioral Neurobiology, Montreal, QC, Canada
**Ventral pallidal encoding of cue-elicited alcohol-seeking behavior**
Jocelyn Richard; Johns Hopkins University, Baltimore, MD, United States
**Preventing relapse to cue-elicited alcohol-seeking with post-retrieval extinction**
Roberto Cofresí; University of Texas, Austin, TX, United States
**Effect of topiramate and aripiprazole on alcohol craving and alcohol consumption in the human laboratory and the natural environment**
Robert Swift; Brown University, Center for Alcohol and Addiction Studies, Providence, RI, United States
**Discussion and Q&A**
Rainer Spanagel; Central Institute of Mental Health, Mannheim, Germany

## Pavlovian 2018

In Fall 2018, Marie and Joanne saw Nadia at the annual meeting of the Pavlovian Society. Marie was invited to organize a symposium, and asked Nadia to be featured as one of the speakers. To-date, this was one of Marie’s favorite symposium to organize. It was entitled “Adjusting to a changing world: individual differences and situation-dependent behaviors in rats and other beasts,” and featured Drs. Nadia Chaudhri, Becca Shansky, Catherine Hartley, and Marie (please see Table 2).

**TABLE 2:** Symposium featuring Drs. Nadia Chaudhri, Becca Shansky, Catherine Hartley, and Marie, presented at the Pavlovian society meeting in Iowa City in 2018.

Adjusting to a changing world: individual differences and situation-dependent behaviors in rats and other beasts
**Pavlovian Society Meeting, Iowa City, 2018**
**Context as a critical cue for alcohol: striatal and amygdala mechanisms**
Nadia Chaudhri; Concordia University, Montreal, Canada
**Tipping the scales: situational modulators of active vs. passive conditioned fear responses**
Becca Shansky; Northeastern University, Boston, United States
**Control and the calibration of motivated behavior**
Catherine Hartley, New York University, New York, United States
**Heterogeneity of extinction phenotypes in rats**
Marie Monfils, University of Texas at Austin, Austin, United States

## Fall 2019: Marie visits Nadia at Concordia University

Dr. Mihaela Iordinova and Nadia invited Marie to visit Concordia University and give a talk. This would be the last time Marie saw Nadia in person. The visit was brief, because a significant weather event in Central Texas disrupted travel and canceled multiple flights the day Marie was set to depart. There was discussion of postponing the trip. Ultimately, Marie ended up taking a red-eye flight to Montreal, with a 2-h hotel nap along the way. Nadia picked Marie up from the airport, and brought her to her Montreal apartment where she fed her breakfast as they chatted and caught up. There, Marie met Moni and Reza, Nadia’s husband and son. Marie still cherishes these moments.

## Spring 2020: Gordon conference on alcohol and the nervous system

Nadia and Rueben were, respectively, invited to attend the prestigious Gordon conference on Alcohol and the nervous system: developing a clearer vision of the integrated neural actions of ethanol, where they had the opportunity to interact, along with their lab members. Nadia’s work was becoming increasingly well-known in the field, and she was fittingly asked to give a presentation. She gave a great talk entitled “Manipulating corticostriatal circuits to curtail reward-related behavior.” Rueben and Nadia welcomed the opportunity to catch up and plan collaborations. Shortly after the meeting, Nadia received her diagnosis.

## Epilogue

We miss Nadia, our collaborator and friend. She departed this world far too soon, and leaves an indelible mark that will endure in our hearts, and our scientific community. Ever since hearing of her diagnosis, we wanted to believe Nadia would be ok, tuning into her personal blog and texting with her throughout the journey. There was an urgency to every exchange. Sadly, in her own words, “the treatments didn’t work.” Throughout it all, Nadia proved to be the force of nature she had already shown us she was. It seems strange, as we write this, that Nadia is not writing alongside us. It still seems she should be on this authorship banner.

## Author contributions

M-HM drafted the first version of this manuscript, edited and approved the final version. HL, RC, and RG edited and approved the final manuscript. All authors contributed to the article and approved the submitted version.
